# Impact of intestinal parasites on microbiota and cobalamin gene sequences: a pilot study

**DOI:** 10.1186/s13071-020-04073-7

**Published:** 2020-04-19

**Authors:** Rojelio Mejia, Ashish Damania, Rebecca Jeun, Patricia E. Bryan, Paola Vargas, Marisa Juarez, Pamela S. Cajal, Julio Nasser, Alejandro Krolewiecki, Emilie Lefoulon, Courtney Long, Evan Drake, Rubén O. Cimino, Barton Slatko

**Affiliations:** 1 National School of Tropical Medicine, Baylor College of Medicine, Houston, TX USA; 2 Universidad Nacional de Salta, Salta, Argentina; 3 New England Biolabs, Inc, Ipswich, MA USA

**Keywords:** *Giardia duodenalis*, Helminths, Cobalamin, Microbiome

## Abstract

**Background:**

Approximately 30% of children worldwide are infected with gastrointestinal parasites. Depending on the species, parasites can disrupt intestinal bacterial microbiota affecting essential vitamin biosynthesis.

**Methods:**

Stool samples were collected from 37 asymptomatic children from a previous cross-sectional Argentinian study. A multi-parallel real-time quantitative PCR was implemented for *Ascaris lumbricoides*, *Ancylostoma duodenale*, *Necator americanus*, *Strongyloides stercoralis*, *Trichuris trichiura*, *Cryptosporidium* spp., *Entamoeba histolytica* and *Giardia duodenalis.* In addition, whole-genome sequencing analysis was conducted for bacterial microbiota on all samples and analyzed using Livermore Metagenomic Analysis Toolkit and DIAMOND software. Separate analyses were carried out for uninfected, *Giardia*-only, *Giardia* + helminth co-infections, and helminth-only groups.

**Results:**

For *Giardia*-only infected children compared to uninfected children, DNA sequencing data showed a decrease in microbiota biodiversity that correlated with increasing *Giardia* burden and was statistically significant using Shannonʼs alpha diversity (*Giardia*-only > 1 fg/µl 2.346; non-infected group 3.253, *P* = 0.0317). An increase in diversity was observed for helminth-only infections with a decrease in diversity for *Giardia* + helminth co-infections (*P* = 0.00178). In *Giardia*-only infections, microbiome taxonomy changed from *Firmicutes* towards increasing proportions of *Prevotella*, with the degree of change related to the intensity of infection compared to uninfected (*P* = 0.0317). The abundance of *Prevotella* bacteria was decreased in the helminths-only group but increased for *Giardia* + helminth co-infections (*P* = 0.0262). Metagenomic analysis determined cobalamin synthesis was decreased in the *Giardia* > 1 fg/µl group compared to both the *Giardia* < 1 fg/µl and the uninfected group (*P* = 0.0369). Giardia + helminth group also had a decrease in cobalamin CbiM genes from helminth-only infections (*P* = 0.000754).

**Conclusion:**

The study results may provide evidence for an effect of parasitic infections enabling the permissive growth of anaerobic bacteria such as *Prevotella*, suggesting an altered capacity of vitamin B12 (cobalamin) biosynthesis and potential impact on growth and development in children 
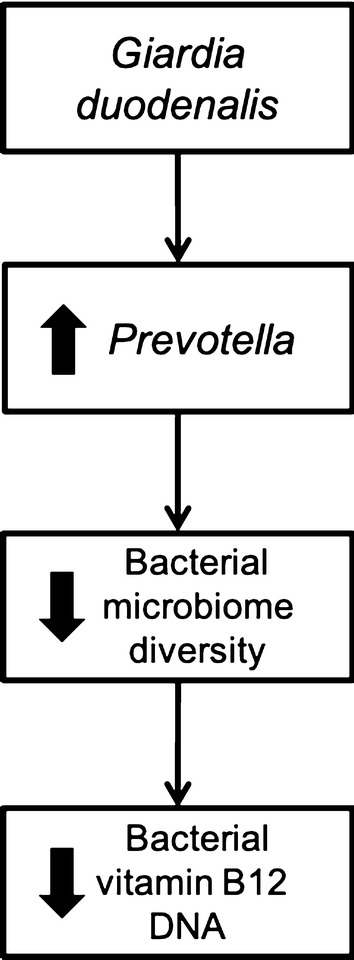
.

## Background

Gastrointestinal (GI) parasites are estimated to infect more than two billion people throughout the world [1]. Both soil-transmitted helminths (STH) (*Ascaris lumbricoides*, hookworms, *Strongyloides stercoralis*, *Trichuris trichiura*) and protozoans (*Giardia duodenalis*, *Cryptosporidium* spp., *Entamoeba histolytica*) are prevalent in resource-limited areas [2, 3]. Symptoms include chronic diarrhea, severe anemia, and can lead to intestinal obstruction. Economically disadvantaged children have recurrent infections and malnutrition that may lead to growth and cognitive delays [4]. These children have more difficulties in school and, subsequently, in the job market [4]. The cycle continues when they remain in poverty and have their children [4]. The link between intestinal helminths and malnutrition leading to growth stunting and anemia has been found by others [5–12], and a Global Burden of Disease Study points to evidence that hookworm is a leading cause of anemia in resource-poor settings [13]. Valuable information from the Global Enteric Multicenter Study (GEMS) and studies related to returning travelers also reveals an unexpected global health impact caused by some protozoans, possibly including giardiasis [14, 15]. The Etiology, Risk Factors, and Interactions of Enteric Infections and Malnutrition and the Consequences for Child Health and Development (MAL-ED) study found an association with subclinical, non-diarrheal giardiasis and decreases in growth of children [16]. There are few studies attributing gut microbiome changes to giardiasis [17–19] and no published studies showing the impact on the human intestinal microbiome using multi-parallel real-time quantitative (qPCR) to detect the presence of *Giardia* and quantitating the burden of infection [20]. To date, most studies examining intestinal parasitism have not been able to study intestinal worms and protozoans simultaneously and to successfully dissect the relative contribution of each of the significant intestinal helminth or protozoan pathogens to specific diseases. The current state of diagnosing gastrointestinal parasites in resource-limited areas uses the subjective method of microscopy. Depending on the parasite, single stool microscopy exam sensitivity ranges from 50–80% [21]. As a result, large numbers of infected children are not being diagnosed correctly and treated.

Gastrointestinal parasites may modulate intestinal inflammation, malabsorption, and microbiome changes [22–26]. The microbiome is associated with digestion, nutrition and health, but alterations in biodiversity can increase disease states and induce intestinal inflammation [27]. Animal studies further show changes in microbial diversity due to *G. duodenalis* [18, 28]. There is a lack of literature studying the relationship between GI parasitesʼ impact on the human intestinal microbiome [19]. These few studies have presented discordant results of the impact parasites have on human intestinal microbiota biodiversity [29, 30].

The relationship between intestinal microbiota biodiversity may depend upon which specific parasite is present in the gut [30]. Since the burden of parasite infection is directly correlated to morbidity and disease, the intensity of infection may also impact the intestinal microbiota [30]. The qPCR quantitates the burden of helminths and protozoans, determining the correlation of burden to changes in intestinal microbiota biodiversity. Alterations in intestinal microbiota alter bacterial metabolites, such as vitamin B12 (cobalamin), reducing their availability for human use.

Interactions between parasites and intestinal microbiota may have a direct impact on child nutrition. *Giardia duodenalis* is known to cause malabsorption, steatorrhea, and diarrhea [31], with preliminary studies finding improvements in vitamin B12 serum levels after treatment for giardiasis [32, 33]. Vitamin B12 is a crucial microbiota-derived co-enzyme for humans who cannot produce it [34–36]. As vitamin B12 production is unique to specific intestinal bacteria, alterations in intestinal microbiota could diminish vitamin B12 availability for human use [37]. Advances in next-generation DNA sequencing allow for precise taxonomic comparisons between intestinal microbiotas and can simultaneously be used to scan the intestinal microbiota meta-genome for the presence of functional genes necessary for the specific functions, like cobalamin synthesis. In this pilot study, parasite qPCR and next-generation DNA sequencing was used to explore whether quantitative burden of specific parasites (*Giardia duodenalis* and soil-transmitted helminths) influence the composition of intestinal microbial communities. Using vitamin B12 as a representative bacteria-generated nutrient, we analyzed bacterial metagenomes as a surrogate for changes in intestinal bacteria functions associated with intestinal parasitic infections. This was a preliminary study using a population with high prevalence of intestinal parasites. It is meant as an introductory for the future direction of our research.

## Methods

### Study population

This descriptive study aimed to determine the effect of *G. duodenalis* and other intestinal parasites on bacterial microbiota and subsequent cobalamin metagenomics. Samples were randomly selected from a previously published study using qPCR in peri-urban Argentina [38]. No previous antiparasitics or antibiotics were administered 3 months prior to the sample collection. Samples consisted of four groups: (i) a control group with no parasites detected by qPCR (uninfected); (ii) a *Giardia*-only infected group; (iii) a *Giardia* and helminth co-infection group; and (iv) a helminth-only infected group. Helminths included in this study were *Ascaris lumbricoides*, *Ancylostoma duodenale*, *Necator americanus* and *Strongyloides stercoralis* (Table 1).

**Table 1:** Metadata for research subjects (geometric mean, minimum, and maximum)

Group (*n*)	Mean age (range) (years)	Male	Female	*Giardia* DNA (fg/µl)	*Ascaris* DNA (fg/µl)	*Ancylostoma* DNA (fg/µl)	*Necator*DNA (fg/µl)	*Strongyloides* DNA (fg/µl)	Shannonʼs alpha diversity mean (range)
Uninfected (*n *= 5)	4.5 (3–6)	3	2	0	0	0	0	0	3.253 (2.826–3.839)
*Giardia* (*n *= 13)	5.6 (4–7)	6	7	1.12 (0.012–20,657)	0	0	0	0	>1 fg/µl = 2.346 (2.066–3.199); < 1 fg/µl = 3.253 (2.250–3.617)
*Giardia *+helminths (*n *= 7)	6.8 (4–8)	4	3	21.4 (0.02–5697.8)	1.062	164.8 (12.95–556.4)	0.249 (0.03–12.47)	39.59 (9.97–157.1)	3.118 (2.201–3.343)
Helminths (*n *= 12)	5.1 (3–7)	6	6	0	6.82 (4.133–9.67)	10627.8 (2320–59963)	2.839 (0.119–63.8)	0.01357	3.407 (3.217–3.783)

*n* number of subjects

### Multi-parallel real-time quantitative PCR

All stools were collected and immediately stored on ice and sealed in air-tight containers, frozen within 3 h, and DNA extracted using MP FastDNA Spin Kits for Soil (MP Biomedicals, Irvine, CA) [38]. This collection method was found to yield adequate microbiome data for study analysis [29]. Samples were processed in Salta, Argentina, using a modified bead-beating process described previously [38, 39]. DNA was processed for qPCR in Houston, Texas, as previously described [38, 39]. The intensity of parasite DNA was calculated using reference plasmids to create a standard curve, as previously reported [38]. The qPCR results from the previous Argentinian study were used for these analyses [38].

### Whole-genome DNA sequencing

DNA sequencing and library construction were performed at New England Biolabs (NEB). All the samples underwent removal of methylated DNA with NEBNext^®^ Microbiome enrichment kits (New England Biolabs, Ipswich, MA). No DNA size selection was made. DNA Library prep was done as per the manufacturerʼs protocol using 1 µg of sample DNA and eight cycles of PCR enrichment (NEBNext Ultra DNA Library Prep Kit for Illumina, Version 5.1, 9/17). Sequencing was performed using an Illumina NextSeq (Illumina, San Diego, CA) with paired or single ends, and 151 bp reads.

### Bioinformatics

Fastq reads were filtered for quality at a minimum Phred score of 20 (99% accuracy) and a minimum length of 50 using Cutadapt version 1.8.3 [40]. Paired-end reads were interleaved using merge_fastq_reads_with_N_separator. Pl Perl script included with Livermore Metagenomics Analysis Toolkit (LMAT) software version 1.2.6 [41]. Fastq files were converted to fasta files using seqtk software version 1.0 (https://github.com/lh3/seqtk). Fasta files were processed by LMAT for taxonomic classification using LMAT kFull database. LMAT output text files were filtered for LMAT defined confidence score of 1 and minimum reads of 500 using tolineage.py script. Subsequently, output files were combined using merge_metaphlan_tables.py script using Metaphlan [42].

Alpha diversity was calculated using the *Phyloseq* R package [43]. Abundant different operational taxonomic units (OTU) among the four groups were identified using the LEfSe algorithm [44]. Most abundant bacteria for Lefse were run with a logarithmic LDA score threshold of 4.5 and other parameters set to default. Metagenomics analysis was performed using Diamond v0.8.4 using blastx mode with 90% minimum identity and e-value of 10^−5^ against nr database fasta file [45]. Results from Diamond analysis were exported to Megan version 6 using daa-meganizer program [46]. GenInfo identifier to Interpro identifier mapping within Megan program was used to annotate the vitamin B12 synthesis gene [47]. STAMP software was used for statistical analysis pertaining to taxonomic and metagenomic differences [48]. Microbial attributes were derived from (LMAT) taxonomic output using the Megan program.

### Statistics

qPCR results were recorded for each patient as positive or negative, including the concentration of DNA (fg/µl) for each parasite. All statistics were performed using Prism v. 7.0b (GraphPad, La Jolla, CA). Mann-Whitney and one-way ANOVA tests were used to compare two and multiple groups, respectively. Spearmanʼs rank test was used to correlate the *Giardia* DNA concentration to Shannonʼs alpha diversity, including the “uninfected” and “*Giardia*-only” group. Shannonʼs alpha diversity is a commonly reported diversity metric that weights the numbers of species by their relative evenness [49]. Proportion of targeted sequences was used in calculating OTU and metagenomics. Vitamin B12 data was calculated comparing specific genes to all other vitamin B12 synthesis genes. All statistical models used *P-*values less than 0.05 as significant.

## Results

### Parasite DNA intensity

Parasite DNA intensity with the intestinal parasite detection by stool qPCR as described by Cimino et al. [38] (Table 1).

### Diversity of intestinal microbiota

**Fig. 1:**
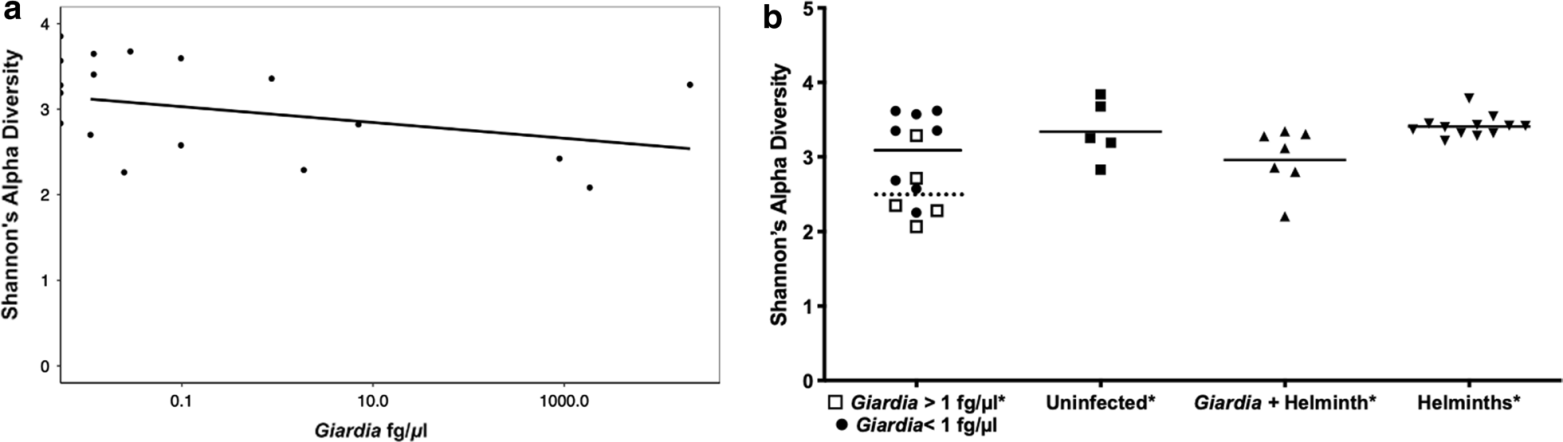
**a** There was an inverse correlation of *Giardia* DNA to decreased Shannonʼs alpha diversity (Spearmanʼs *r* = − 0.4781, *P* = 0.0447); > 1 fg/µl was selected as heavy *Giardia* DNA burden since a noticeable change in slope occurred after 1 fg/µl. **b** The mean of the *Giardia* > 1 fg/µl group (dotted line) has a significant decrease in bacterial diversity compared with the uninfected group (*P* = 0.0317) and all other groups (*P* = 0.000166). *P < 0.05

Measuring bacterial diversity in relation from children with no *Giardia* DNA to increasing *Giardia* DNA intensity of burden (fg/µl) showed an inverse correlation of bacterial diversity to *Giardia* DNA (fg/µl) (Spearmanʼs *r*_(18)_ = − 0.4781, *P* = 0.0447) (Fig. 1a). From the scatter plot (Fig. 1a) a noticeable change in slope occurred at levels > 1 fg/µl. Since there are no guidelines for heavy *Giardia* burden, greater than 1 fg/µl was selected, and all further analyses validated this threshold.

*Giardia* > 1 fg/µl group had a significantly lower biodiversity compared to the uninfected children using Shannonʼs alpha diversity (*Giardia*-only > 1 fg/µl: 2.346; uninfected group: 3.253, *U*_(10)_ = 2, *Z* = − 2.1934, *P* = 0.0317 (Fig. 1b). *Giardia* > 1 fg/µl had lower diversity than the helminth-only group *U*_(17)_ = 0, *Z* = − 3.1096, *P* = 0.0003 (Fig. 1b). *Giardia* > 1 fg/µl also had lower diversity compared to all other groups *F*_(3, 25)_ = 9.982, *P* = 0.000166 (Fig. 1b). Alpha diversity was also lower for the *Giardia* + helminth co-infected group compared to the helminth-only group *U*_(19)_ = 7, *Z* = 2.958, *P* = 0.00178 (Fig. 1b).

### Change in bacterial abundance

**Fig. 2:**
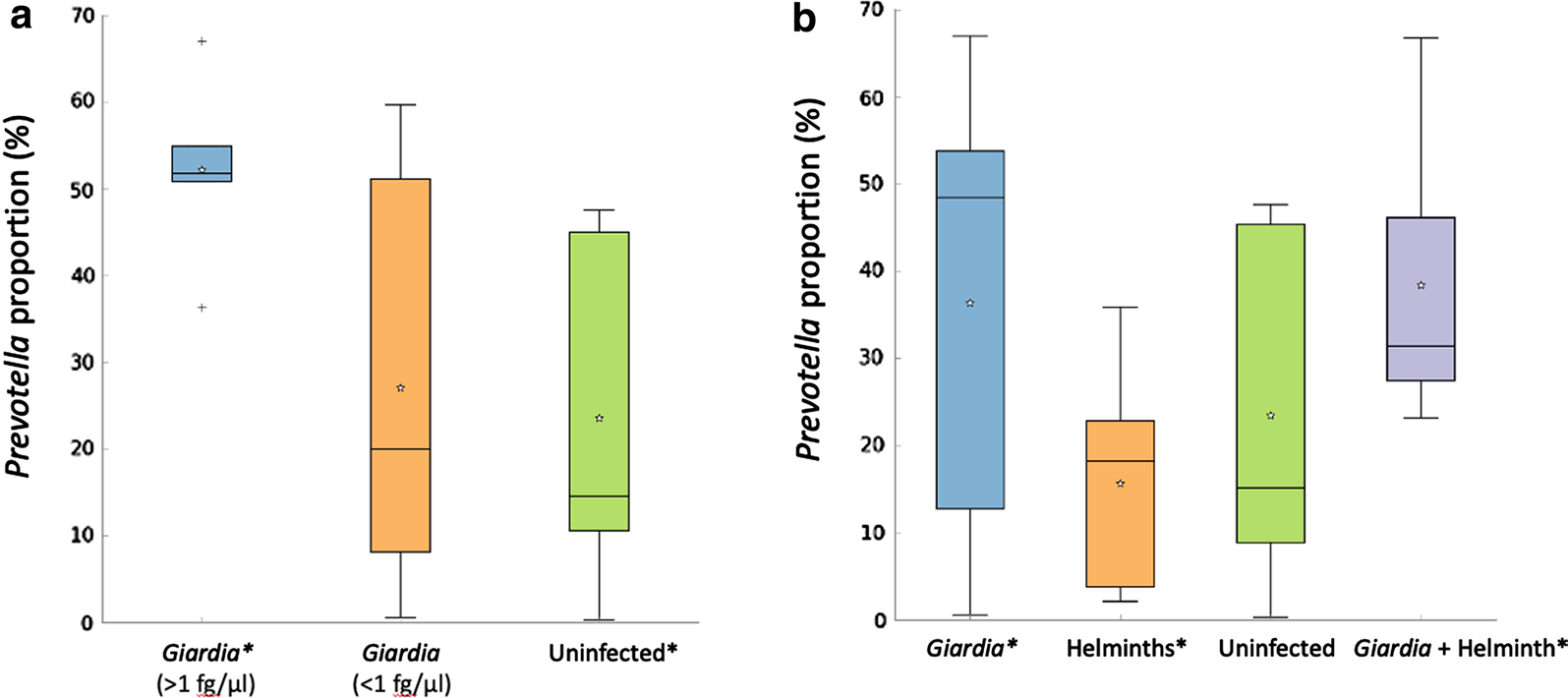
**a***Giardia* > 1 fg/µl group had greater proportions of *Prevotella* DNA than the uninfected group (*P* = 0.0317) in their intestinal microbiota. **b** The helminths-only group had decreased proportions of *Prevotella* DNA compared with the *Giardia*-only group (*P* = 0.0457) in their intestinal microbiota. The *Giardia* + helminth co-infected group had increased proportion of *Prevotella* DNA compared to the helminth-only group (*P* = 0.00262) in their intestinal microbiota. *P < 0.05

In *Giardia-*only infections, microbiome analysis data indicates decreased biodiversity in the infected parasite group compared to the non-infected group, a bias toward increased *Prevotella*, with the degree of change related to the intensity of infection (Fig. 2a). G*iardia*-only children had significantly higher proportions of the genus *Prevotella* bacteria directly correlating to above 1 fg/µl *Giardia* DNA *versus* uninfected group *U*_(10)_ = 2, *Z* = 2.1934, *P* = 0.0317 (Fig. 2a). The helminth-only group with decreased *Prevotella* proportions relative to the *Giardia-*only group *U*_(25)_ = 41, *Z* = 2.0125, *P* = 0.0457, but similar to the control group (Fig. 2b). Interestingly, the *Giardia* + helminth co-infected group had increased *Prevotella* proportions compared to the helminth-only group *U*_(19)_ = 8, *Z* = − 2.8735, *P* = 0.00262 (Fig. 2b). *Prevotella copri* was the major species in each group, *Giardia* (37%), helminths-only (17%), uninfected (22%), *Giardia* + helminths (36%) (data not shown).

**Fig. 3:**
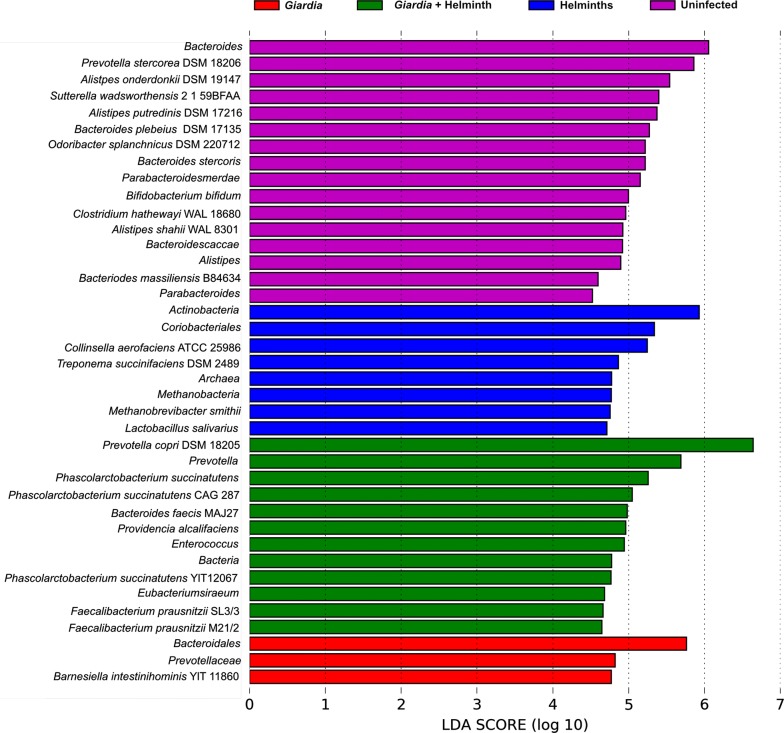
Most abundant OTUs for each group using LDA Effect Size (LEfSe)

All four groups had different bacteria genera as their most abundant microbiota. *Giardia-* infected children, including *Giardia* + helminth co-infected, had higher Bacteroidales, including *Prevotella* species (Fig. 3). Specific cobalamin producing bacteria, such as *Lactobacillus* and *Bifidobacterium*, were found in higher abundance for the non-*Giardia* infected children (Fig. 3).

### Metagenomics of cobalamin biosynthesis

**Fig. 4:**
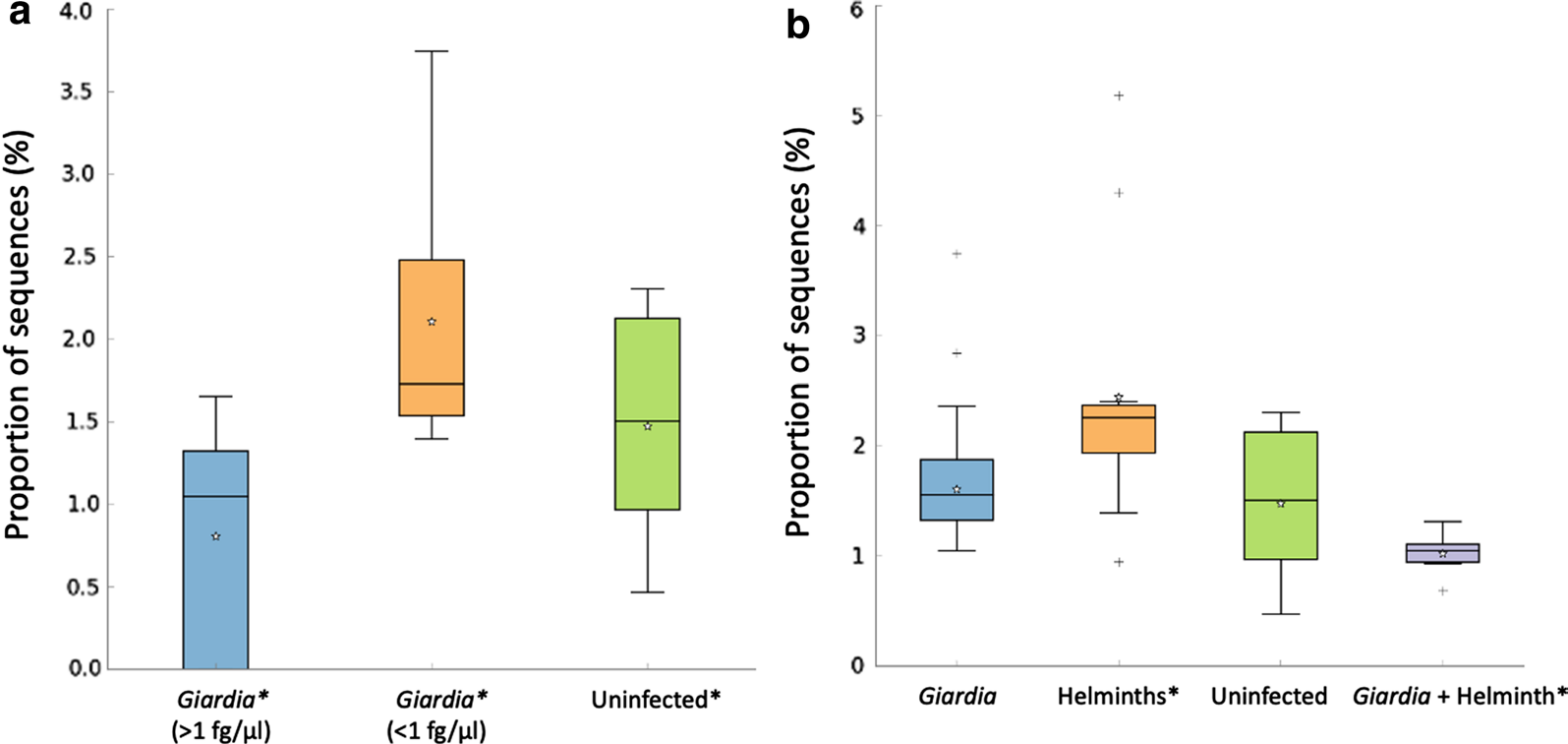
**a** Children infected with > 1 fg/µl *Giardia* DNA concentrations had decreased cobalamin biosynthesis genes compared to the combined *Giardia* < 1  fg/µl and uninfected groups (*P *= 0.0369) with **b** the Giardia + helminth group having less cobalamin biosynthesis genes than the helminth-only infections (*P *= 0.000754). *P < 0.05

**Fig. 5:**
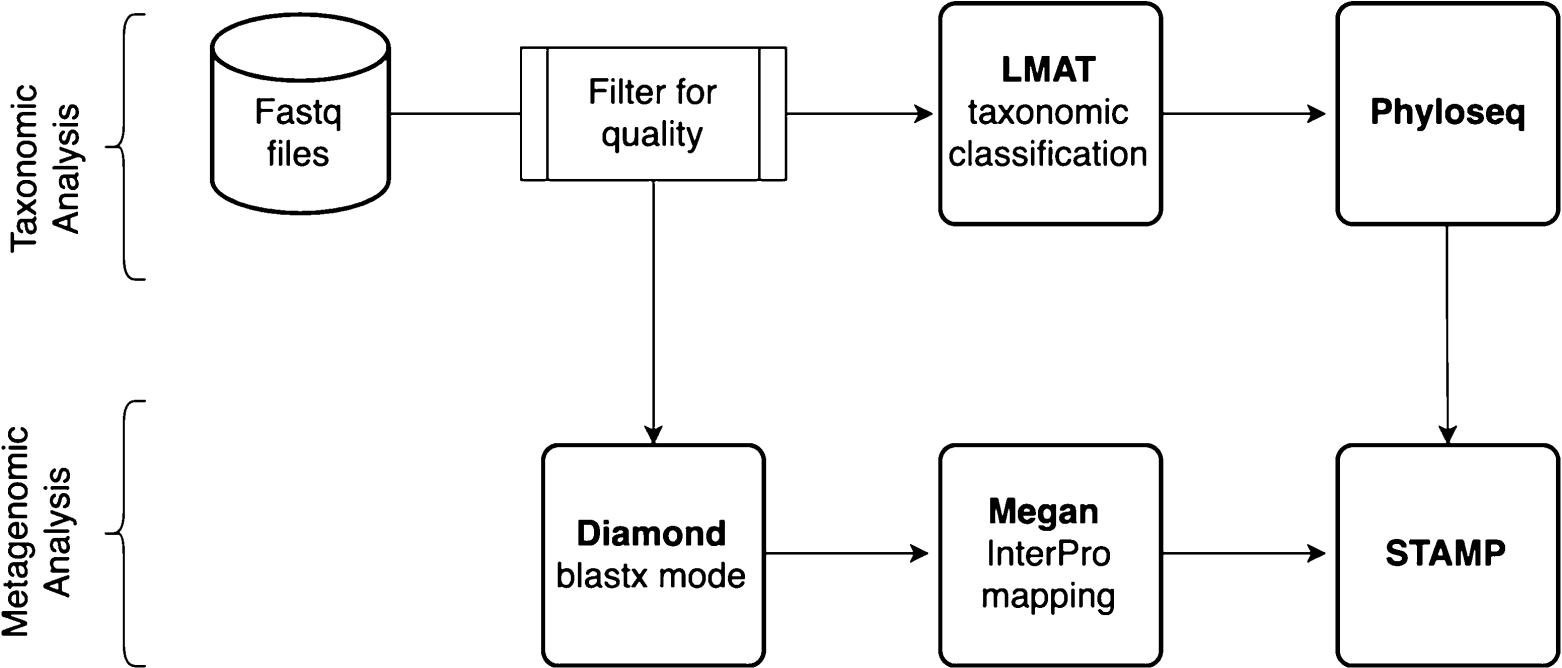
Flowchart for bioinformatics and data processing

Vitamin B12 InterPro identifier IPR002751 biosynthesis CbiM gene was used in the analysis. Children with *Giardia* > 1 fg/µl infections had fewer quantities of cobalamin DNA sequences than the *Giardia* < 1 fg/µl and uninfected group combined *F*_(2, 15)_ = 4.145, *P* = 0.0369 (Fig. 4a). Children with *Giardia* + helminth co-infections had a less proportion of vitamin B12 pathway DNA sequences, compared to helminth-group *U*_(19)_ = 5, *Z* = 3.1271, *P* = 0.000754 (Figs. 4b, 5).

## Discussion

The association of *G. duodenalis* with microbiome diversity was observed in this study using whole-genome sequencing. *Giardia duodenalis* plays a prominent role, perhaps as its primary site of infection and replication is in the small intestine.

### Parasites alter the intestinal microbiota of children

This study determined that children infected with *Giardia* DNA > 1 fg/µl are associated with decreased microbial diversity and increases of *Prevotella.* DNA levels of *Giardia* > 1 fg/µl implies that there are more parasites to alter the intestinal microbiome, and thus have a higher impact on intestinal bacterial species.

It is unclear whether the parasites are impacting the intestinal microbiota or external factors such as age, diet, or sex differences altering the intestinal microbiota and making the subjects more susceptible to enabling a *G. duodenalis* infection. Certain bacteria can permit *G. duodenalis* colonization, as evidenced from a mouse model study where the mouse intestinal microbiota (enteroaggregative *Escherichia coli*), independent of *G. duodenalis* infection, can promote inflammation, but together synergistically increased signals of intestinal injury [18].

### Changes in vitamin B12 due to parasite infections

Vitamin B12 synthesis primarily occurs in anaerobes, including *Bifidobacterium* and *Lactobacillus* species [37, 50–53]. These microorganisms may promote intestinal homeostasis and may protect against inflammatory diseases [54–57]. Vitamin B12 is absorbed in the small intestines [58–60] while the majority of microbiota reside in the colon [58], although, the small intestine is not sterile and does contain a robust microbiota that influences the absorption of vitamins [61–63]. Specific bacteria produce vitamin B12 [64], and the children infected with *G. duodenalis* with DNA levels above 1 fg/µl may be unable to synthesize the required amounts of vitamin B12 for nutritional benefit. The group with less than 1 fg/µl *Giardia* DNA infections had equal vitamin B12 bacterial genes to the uninfected group, both being higher than the *Giardia* > 1 fg/µl group, likely showing that lower intensity of *Giardia* infections has similar effects as the uninfected group on vitamin B12 synthesis.

Evidence of the impact of *G. duodenalis* on the diversity and available micronutrients was also observed in the *Giardia* + helminth co-infection group, compared to the alterations of the microbiome seen in the helminth-only group. While the helminth-only group did not have changes in diversity or decreased cobalamin synthesis genes, a possible explanation is that most of these helminths reside in the colon and do not alter the microenvironments as does *G. duodenalis*.

### Study limitations

A limitation of this cross-sectional study is the small sample size. However, the results are consistent, and this potential limitation can be viewed in terms of the sheer number, and fidelity of, using enriched microbe DNA for whole gene sequencing, producing millions of reads for analysis. This study also did not take into account *Giardia* assemblages that can have differing amount of pathogenicity. Another limitation is that serum vitamin B12 levels were not measures in children and thus the decrease of vitamin B12 genes in *Giardia* infected children could not be translated into a loss of vitamin B12.

## Conclusions

In this study, there is a possible link as to why *G. duodenalis* and other parasites may cause growth and developmental delays in infected children. *Giardia-*infected children with > 1 fg/µl DNA concentrations were associated with less microbiome diversity, and a higher abundance of *Prevotella* associated with the diminished presence of cobalamin synthesis genes. The influence of *G. duodenalis* appears to be evident regardless of the presence of STH, and was associated with altered microbiome composition or function as measured by cobalamin synthesis. This descriptive study is a preliminary evidence for future studies looking at the cobalamin biosynthesis pathway. We are currently extending these results to additional populations where *G. duodenalis* and other intestinal parasite infections are endemic, determining *Giardia* assemblages, and confirming the biochemical analysis of the vitamin B12 pathway products in affected children.

## Data Availability

The data supporting the conclusions of this article are available in the NCBI BioProject repository, accession PRJNA612291 https://www.ncbi.nlm.nih.gov/bioproject/PRJNA612291/.

## Abbreviations

DNA: Deoxyribonucleic acid
GEMS: Global enteric multicenter study
GI: Gastrointestinal
LMAT: Livermore metagenomics analysis toolkit
OTU: Operational taxonomic unit
qPCR: Multi-parallel real-time quantitative polymerase chain reaction
STH: Soil-transmitted helminths
